# Supplementary material for: Dissolved organic matter and sulfide enhance the CH4 consumption of a psychrophilic lake methanotroph, Methylobacter sp. S3L5C
Source: Microbiol Spectr. 2025 Jun 9;13(7):e03133-24. doi: 10.1128/spectrum.03133-24 (PMC12211021; doi:10.1128/spectrum.03133-24)
Supplement: Supplemental materials — Additional methods and Fig. S1 to S4. [file spectrum.03133-24-s0001.pdf]

Supplementary data includes:

1. Supplementary methods (included in this .pdf file)
2. Supplementary Figures S1-S4 (included in this .pdf file)
3. Supplementary Table S1 (Supplementary_TableS1.xlxs)
4. Supplementary dataset on CH_4, CO_2, OD_{600}, thiosulfate and sulfate (Gas_OD_S_data.xlsx)

1. Supplementary methods

1.1 Culture and medium

A psychrophilic *Methylobacter* sp. S3L5C (Khanongnuch et al., 2022) is available at the Laboratory of Bio and Circular Economy research group, Materials Science and Environmental Engineering, Tampere University, Finland. This study employed a nitrate mineral salts (NMS) medium modified from DSMZ medium 921 with pH of ~6.8 containing, per liter, 1 g KNO_3 (10.0 mM), 1 g $MgSO_4 \cdot 7H_2O$ (4.0 mM), $CaCl_2 \cdot 2H_2O$ (1.4 mM), 0.35 g K_2HPO_4 (2 mM), 0.28 g KH_2PO_4 (2 mM), 1 mL of 11 mM Fe(III)-EDTA, 1 mL of 0.7 mM $LaCl_3$, 1 mL of 1 mM $Na_2MoO_4 \cdot 2H_2O$ and 1 mL trace elements solution. The latter comprises of, per liter, 1 g $CuSO_4 \cdot 5H_2O$, 0.5 g $FeSO_4 \cdot 7H_2O$, 0.4 g $ZnSO_4 \cdot 7H_2O$, 0.015 g H_3BO_3, 0.05 g $CoCl_2 \cdot 6H_2O$, 0.02 g $MnCl \cdot 4H_2O$, and 0.01 g $NiCl_2 \cdot {}_6H_2O$. Before conducting each experiment, *Methylobacter* sp. S3L5C was pre-cultivated and maintained as an active culture in 150 mL NMS in 500 mL glass bottles closed with rubber stoppers and aluminum screw caps with a central hole. 60 mL of headspace was replaced with CH_4 to create a mixture of 20 vol% CH_4 and 80 vol% air. In this work, CH_4 was fed into the bottle through butyl rubber stoppers using a Luer-Lock syringe (20 mL, Terumo Corporation, Japan or 50

mL BD Plastipak) equipped with a 25G × 1” BD Microlance™ 3 needle (Becton, Dickinson, USA). All procedures were conducted under sterile conditions.

1.2 Effect of different sulfide concentrations on the growth of *Methylobacter* sp. S3L5C

The cell pellets of *Methylobacter* sp. S3L5C were harvested at the mid-exponential growth phase of the pre-cultured bottles and inoculated in fresh 5 mL NMS medium in 25 mL cultivation tubes (18 in diameter × 150 mm in length) equipped with grey rubber stopper (Chromacol 20-B3P, Germany) and aluminum crimp seal. After sealing, sterile filtered 100 mM Na_2S solution was transferred and introduced to each tube using a 1 mL Luer-Lock syringe (Terumo Corporation, Japan) equipped with a 25G × 1” BD Microlance™ 3 needle (Becton, Dickinson, USA) to obtain different sulfide concentrations of 0, 0.05, 0.1, 0.5, 1, and 5 mM. 20 vol% of headspace was replaced with CH_4. The vials were placed horizontally and incubated under static conditions in a dark, cold room at 5 ± 2 °C for 8 days. The growth was monitored daily by measuring optical density through the tube. The addition of Na2S was not shown to increase pH of the medium at sulfide concentrations 0-1 mM, i.e., pH stayed at ~6.8, while a pH increase was observed at 5 mM sulfide, when pH was ~7.3, which, however, is also within the optimal pH range for the growth of S3L5C (6.0-7.3, see Khanongnuch et al. 2022).

1.3 Effect of freshwater DOM and sulfide on the metabolism of Methylobacter sp. S3L5C

In this test, *Methylobacter* sp. S3L5C was grown in 40 ml NMS medium in 160 ml serum bottles sealed with butyl rubber stoppers and aluminum crimps. The effect of DOM and sulfide on the metabolism of *Methylobacter* sp. S3L5C was evaluated under both high CH_4 concentration (20 vol%) and CH_4-limited conditions (1 vol%), with three different treatments: (i) addition of freshwater DOM alone (at 60 mg L^{-1}), (ii) addition of both DOM

(at 60 mg L^{-1}) and sulfide (at 0.05 mM) and (iii) without any compound addition. All tests were conducted in biological triplicate and in parallel with abiotic controls (sterile medium without cells). The bottles were incubated in a KS 4000 i control incubator (IKA, Germany) with a temperature set at 6 ± 1.5 °C and shaking at 120 rpm, located in the cold room. We periodically monitored growth, pH, dissolved O_2, sulfate, and thiosulfate in a liquid medium, and CH_4 and CO_2 composition in the headspace. Cell pellets were harvested for RNA extraction to study mRNA expression patterns at the mid-exponential growth phase at 80 h for all the tests except the incubation with only 20 vol% CH_4 at 95 h (Fig. 1D).

The applied DOM product was isolated by reverse osmosis combined with electrodialysis from Upper Mississippi river (1R110N, International Humic Substances Society, C% = 49.98 and N% = 2.36). The applied concentration corresponds to ~30 mg L^{-1} of dissolved organic carbon. Based on our further characterization of the elemental composition of DOM (see Section 1.4) the added DOM corresponded also to ~0.65 µmol L^{-1} of iron but negligible concentration of manganese.

1.4 Analytical methods

Regarding the cultivation of *Methylobacter* sp. S3L5C in 25-mL cultivation tubes in different sulfide concentrations experiment (Section 1.2), the growth was measured using an Ultrospec 500 pro spectrophotometer (Amersham Biosciences, UK) at an absorbance wavelength of 600 nm. For liquid sampling in DOM and sulfide experiment (Section 1.3), the liquid culture (1 mL) was withdrawn from batch bottles using a 2-ml BD Discardit™ II syringe equipped with a 25G × 1" BD Microlance™ 3 needle (Becton, Dickinson, USA). The pH was directly measured using a pH 330i portable meter (WTW, Germany) equipped with SlimTrode™ pH electrode (Hamilton, Switzerland), and optical density was measured using a UV-19000i spectrophotometer (Shimadzu Corporation, Japan) at an absorbance wavelength of 600 nm, using a semi-micro disposable cuvette. Then, the liquid culture was centrifuged (at 2700 ×g)

to collect the supernatant, which was subsequently filtered through a 0.2 μm Chromafil® Xtra PET 20/25 syringe filter (Macherey-Nagel, Germany). The filtered liquid was stored at -20 °C for further anion quantitative analysis. Sulfate and thiosulfate were measured using Dionex ICS-1600 ion chromatograph equipped with Dionex IonPac™ AS22 4×250 mm analytical column (Thermo Scientific, USA) and the DIONEX AS-DV autosampler (Thermo Fisher Scientific, USA) using the eluent containing the mixture of 4.8 mM Na_2CO_3 and 1 mM $NaHCO_3$. The elemental composition of the DOM was…

CH_4 and CO_2 were measured using a Shimadzu GC-2014 gas chromatography-thermal conductivity detector (Shimadzu Corporation, Japan) equipped with Porapak N 80-100 (1.8 m length × 2.0 mm diameter) (Agilent Technologies, USA). Briefly, helium was used as the carrier gas at 25 mL min^{-1}, and the column temperature was 40 °C with a hold time of 3.3 min, while the injector and detector temperatures were 80 °C.

Dissolved O_2 of liquid phase was measured nondestructively inside the experimental bottles of DOM and sulfide experiment (Section 1.3) for treatments without DOM or sulfide and for treatments with addition of DOM+sulfide using autoclaved PSt6 sensors (O_2 measurement range: 0 – 2 mg L^{-1}) and OXY-1 SMA trace Single Channel Fiber Optic oxygen transmitter (PreSens Precision Sensing GmbH, Regensburg, Germany).

Further elemental characterization of the Upper Mississippi river DOM was made using inductively coupled plasma mass spectrometry (ICP-MS). For ICP-MS analysis, the DOM samples (79.5±0.64 mg dry weight, n = 2) were decomposed by microwave assisted acid digestion [8 mL of 67-69% (w/w) HNO_3 and 2 mL 30% (w/w) H_2O_2; modified from Gottschalch et al., 2007, CEM 2018]. Acid digestion was conducted for 15 min at 200°C with MARS6 (CEM Corporation, Matthews, NC, USA). Elemental concentrations of filtered (0.20 μm) and diluted acid digestates were measured with ICP-MS (Thermo Scientific iCAP RQ,

Thermo Fisher Scientific Inc., Waltham, MA, USA) with (28Si, 54Fe, 55Mn, 59Co, 62Ni) or without (44Ca, 47Ti) helium as collision gas, and 45Sc and 73Ge as internal standards. The concentrations (µg/g, average ± SD, n=2) of Fe, Mn, Si, Ca, Ti, Co, and Ni were 604.9 ± 41.1, 0, 63.7 ± 2.9, 405.4 ± 21.0, 4.4 ± 0.04, 0.66 ± 0.04, and 10.4 ± 0.7, respectively.

1.5 RNA extraction and data analysis

To collect cell pellets, 35 mL liquid culture from individual bottle (DOM and sulfide experiment; Section 1.3) was centrifuged at 500 rpm (Sigma 4-16KA, Sigma, Germany) for 5 min and washed using fresh NMS to wash extracellular compounds in a liquid medium. The cell pellets were then submerged in RNAlater (Thermo Fisher Scientific, USA) and stored at -20°C until further analysis. RNA extraction, library preparation, and sequencing were performed by Biomarker Technologies (BMK) GmbH (Münster, Germany). Briefly, prokaryotic mRNA library preparation was performed with rRNA depletion, followed by sequencing on the Illumina NovaSeq 6000 platform, generating 150 bp pair-end reads as raw data.

The raw reads were quality-controlled using FastQC v.0.12.1 (https://www.bioinformatics.babraham.ac.uk/projects/fastqc/; accessed 02.09.2024) with MultiQC v.1.24.1 (Ewels et al., 2016) for summarization. The reads were subsequently aligned to the *Methylobacter* sp. S3L5C genome (GenBank CP076024.1; Khanongnuch et al., 2022) using Bowtie2 v.2.5.4 (https://bowtie-bio.sourceforge.net/bowtie2/index.shtml.; accessed 02.09.2024) (Langmead and Salzberg, 2012) . The genome was functionally annotated by NCBI Prokaryotic Genome Annotation Pipeline (PGAP) (https://www.ncbi.nlm.nih.gov/genome/annotation_prok/, May 27, 2021) (Tatusova et al., 2016) and available from NCBI with accession number GCA_022788635.1. Genes encoding extracellular electron transfer, e.g. iron/DOM oxidation genes, were discovered using FeGenie, a bioinformatic tool for identification of iron-related genes and gene neighborhood

(https://github.com/Arkadiy-Garber/FeGenie) (Garber et al., 2020). The alignment quality, e.g., strandness and parameters for counting, was evaluated using RSeQC v5.0.1 (https://rseqc.sourceforge.net/; accessed 05.09.2024) (Wang et al., 2012). The number of reads mapped to each gene was counted using HTseq-count v.2.0.5 (Putri et al., 2022) . The raw HTseq counts assigned with genes were further normalized and differentially expressed genes were analysed using the R/Bioconductor package DESeq2 (Anders and Huber, 2010). Significantly differential mRNA expression among different treatments were tested using Wald test with multiple testing correction using the false discovery rate (FDR) controlling method of Benjamini and Hochberg, the default statistical test for differential expression in DEseq2. Then, adaptive shrinkage was used to enhance estimation of FDR and true effect sizes (e.g. fold change in gene expression) using the R package ashr (http://github.com/stephens999/ashr) (Stephens, 2017). The trimming of raw reads was omitted as it could remove important genes from the dataset(Liao and Shi, 2020).

1.6 Statistical analysis

Repeated measures ANOVA was used to test the differences in OD_{600} and concentrations of CH_4 and CO_2 between treatments. P value of <0.05 was considered statistically significant. In case of statistically significant interaction between time and the tested variable, the effect of treatment was tested at each time point followed by pairwise comparisons using Bonferroni corrected p-values. The analysis was conducted in R (v. 4.2.2) (R Core Team, 2021) operated in RStudio (v. 2024.09.1) (RStudio Team, 2020) using packages rstatix (v. 0.7.2) (Kassambara, 2023a), dplyr (v. 1.1.4) (Wickham et al., 2023), tidyverse (v. 2.0.0) (Wickham et al., 2019), and ggpubr (v. 0.6.0) (Kassambara, 2023b).

Supplementary reference

Anders, S., Huber, W., 2010. Differential expression analysis for sequence count data. Genome Biol. 11, R106. https://doi.org/10.1186/gb-2010-11-10-r106

CEM. 2018. MARS 6 Microwave Acid Digestion. Method Note Compendium. CEM Corporation, Matthews, NC, USA.

Ewels, P., Magnusson, M., Lundin, S., Käller, M., 2016. MultiQC: summarize analysis results for multiple tools and samples in a single report. Bioinformatics 32, 3047–3048. https://doi.org/10.1093/bioinformatics/btw354

Garber, A.I., Nealson, K.H., Okamoto, A., McAllister, S.M., Chan, C.S., Barco, R.A., Merino, N., 2020. FeGenie: A Comprehensive Tool for the Identification of Iron Genes and Iron Gene Neighborhoods in Genome and Metagenome Assemblies. Front. Microbiol. 11. https://doi.org/10.3389/fmicb.2020.00037

Gottschalch, U., Birke, M., Kupsch, H., Stärk, H.-J., Lippold, H. 2007. Characterization of urban NOM in a municipal area with disused toxic waste sites. Applied Geochemistry 22, 2435-2455. doi:10.1016/j.apgeochem.2007.06.014

Kassambara, A., 2023a. rstatix: Pipe-Friendly Framework for Basic Statistical Tests. R package version 0.7.2, https://rpkgs.datanovia.com/rstatix/.

Kassambara, A., 2023b. ggpubr: ‘ggplot2’ Based Publication Ready Plots. R package version 0.6.0, https://rpkgs.datanovia.com/ggpubr/.

Khanongnuch, R., Mangayil, R., Svenning, M.M., Rissanen, A.J., 2022. Characterization and genome analysis of a psychrophilic methanotroph representing a ubiquitous Methylobacter spp. cluster in boreal lake ecosystems. ISME Commun. 2, 85. https://doi.org/10.1038/s43705-022-00172-x

Langmead, B., Salzberg, S.L., 2012. Fast gapped-read alignment with Bowtie 2. Nat. Methods 9, 357–359. https://doi.org/10.1038/nmeth.1923

Liao, Y., Shi, W., 2020. Read trimming is not required for mapping and quantification of RNA-seq reads at the gene level. NAR Genomics Bioinforma. 2, lqaa068. https://doi.org/10.1093/nargab/lqaa068

Putri, G.H., Anders, S., Pyl, P.T., Pimanda, J.E., Zanini, F., 2022. Analysing high-throughput sequencing data in Python with HTSeq 2.0. Bioinformatics 38, 2943–2945. https://doi.org/10.1093/bioinformatics/btac166

R Core Team, 2021. R: A language and environment for statistical computing. R Foundation for Statistical Computing, Vienna, Austria. https://www.R-project.org/.

Stephens, M., 2017. False discovery rates: a new deal. Biostatistics 18, 275–294. https://doi.org/10.1093/biostatistics/kxw041

Tatusova, T., Dicuccio, M., Badretdin, A., Chetvernin, V., Nawrocki, E.P., Zaslavsky, L., Lomsadze, A., Pruitt, K.D., Borodovsky, M., Ostell, J., 2016. NCBI prokaryotic genome annotation pipeline. Nucleic Acids Res. 44, 6614–6624. https://doi.org/10.1093/nar/gkw569

Wang, L., Wang, S., Li, W., 2012. RSeQC: quality control of RNA-seq experiments. Bioinformatics 28, 2184–2185. https://doi.org/10.1093/bioinformatics/bts356

Wickham, H., Averick, M., Bryan, J., Chang, W., McGowan, L.D., François, R., Grolemund, G., Hayes, A., Henry, L., Hester, J., Kuhn, M., Pedersen, T.L., Miller, E., Bache, S.M., Müller, K., Ooms, J., Robinson, D., Seidel, D.P., Spinu, V., Takahashi, K., Vaughan, D., Wilke, C., Woo, K., Yutani, H., 2019. Welcome to the Tidyverse. J. Open Source Softw. 4, 1686. https://doi.org/10.21105/joss.01686

Wickham, H., François, R., Henry, L., Müller, K., Vaughan, D., 2023. dplyr: A Grammar of Data Manipulation. R package version 1.1.4, https://github.com/tidyverse/dplyr, https://dplyr.tidyverse.org.

Figure S1

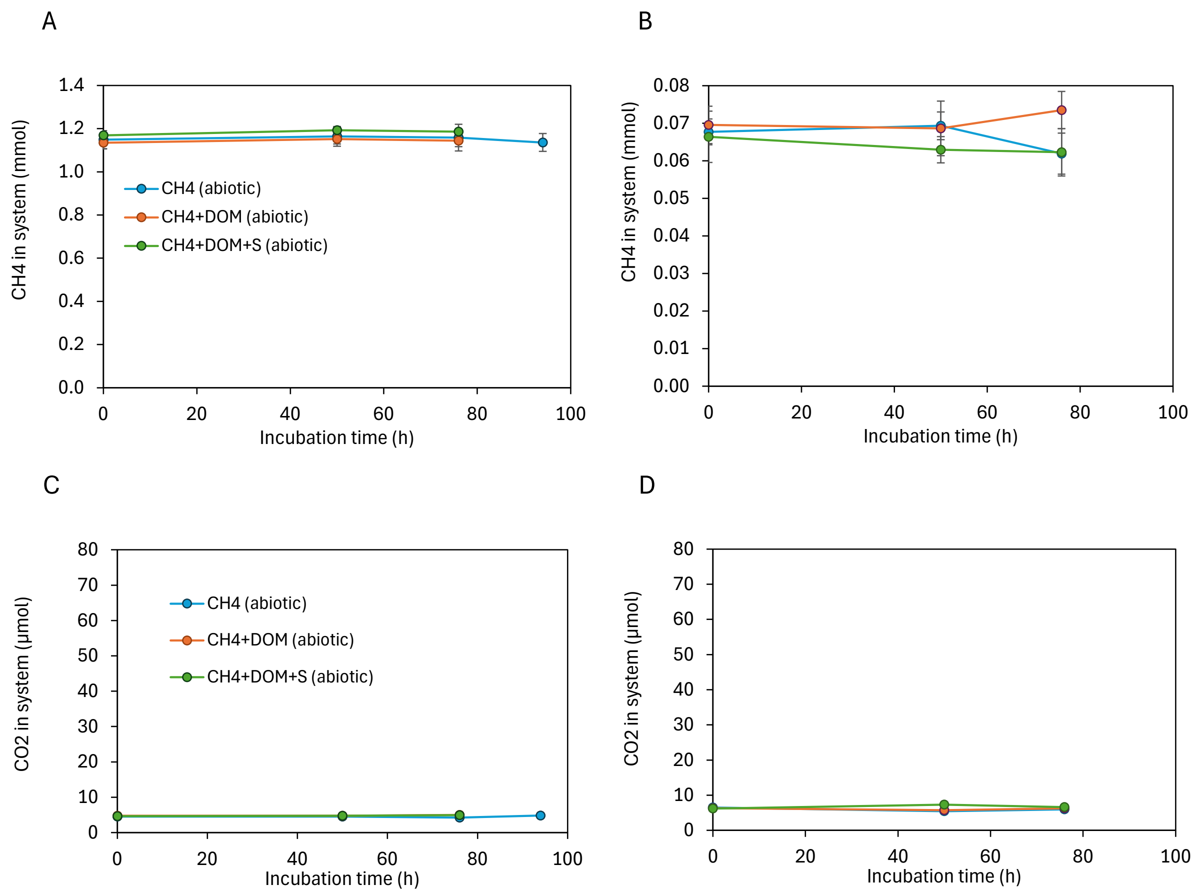

Figure S1. A,B) CH_4 and C,D) CO_2 concentrations in abiotic controls (sterile medium without cells) at initial 20%CH_4 (A,C) and 1%CH_4 (B,D). Treatments are 1. CH_4, 2. CH_4 + dissolved organic matter (DOM) and 3. CH_4 + DOM + sulfide. CH_4 consumption or CO_2 production was not detected in abiotic controls.

Figure S2

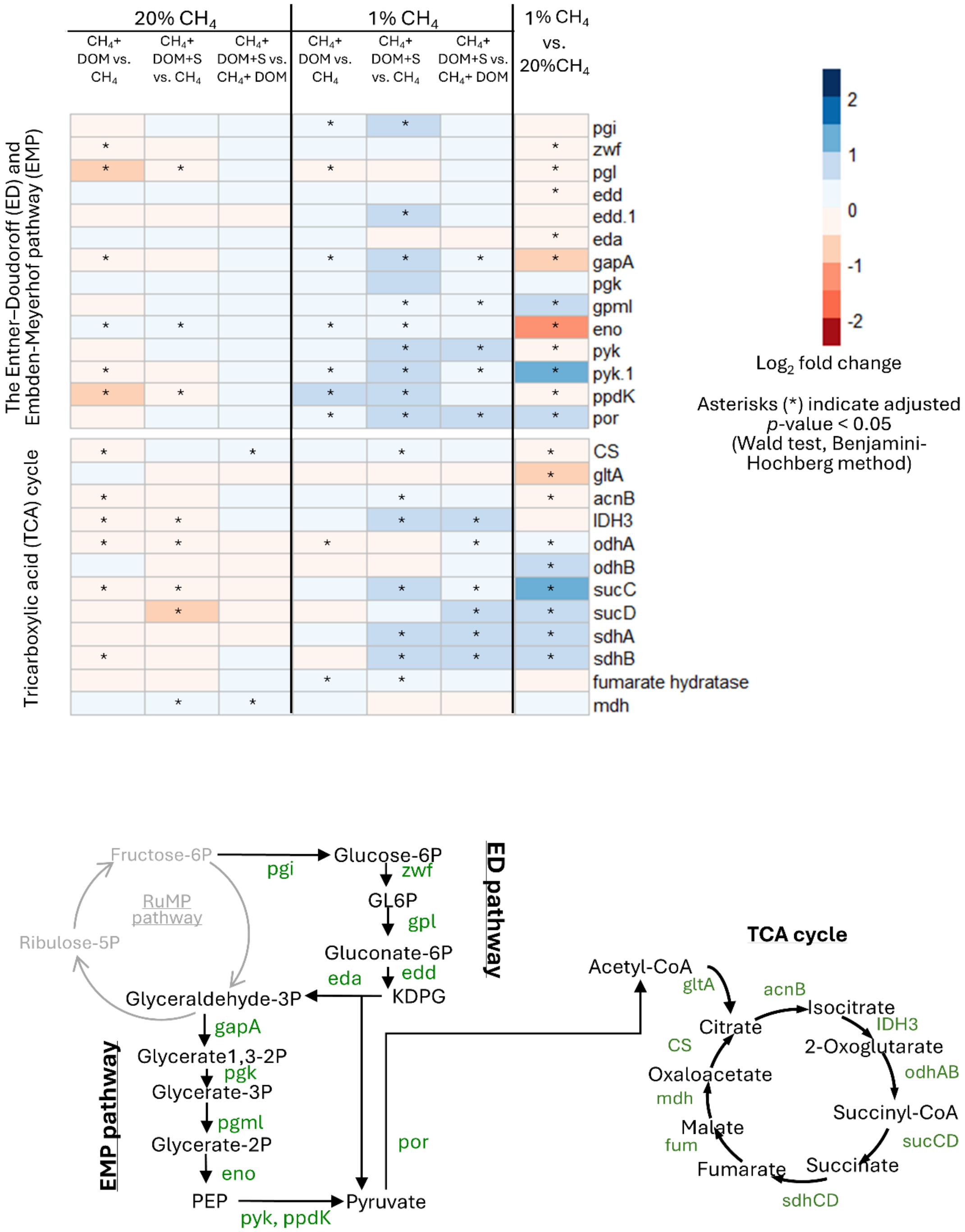

Figure S2. Differential expression of genes involving Embden-Meyerhof-Parnas (EMP) and Entner–Doudoroff (ED) pathways and tricarboxylic acid (TCA) cycle, compared across different treatments: 1. CH_4 + DOM vs. CH_4, 2. CH_4+DOM+S vs. CH_4, 3. CH_4 + DOM + S vs. CH_4 + DOM at initial 20% CH_4 and 1% CH_4, as well as 4. initial 1% CH_4 vs. initial 20%

CH_4. Asterisks indicate significant log2fold change. See full details on gene annotations, normalized counts, and DESeq2 analyses in Supplementary Table S1.

Figure S3

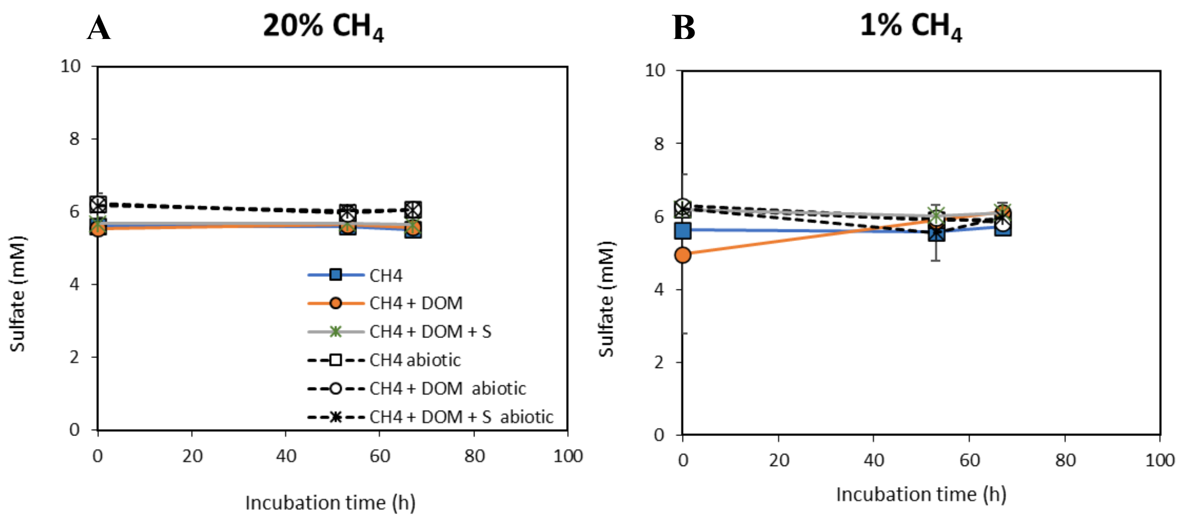

Figure S3. Sulfate concentration in the liquid phase during the experiment testing the effect of dissolved organic matter (DOM) and sulfide (S) on CH_4 metabolism of *Methylobacter* sp. S3L5C at initial A) 20%CH_4 and B) 1%CH_4 in headspace.

Supplementary Figure S4

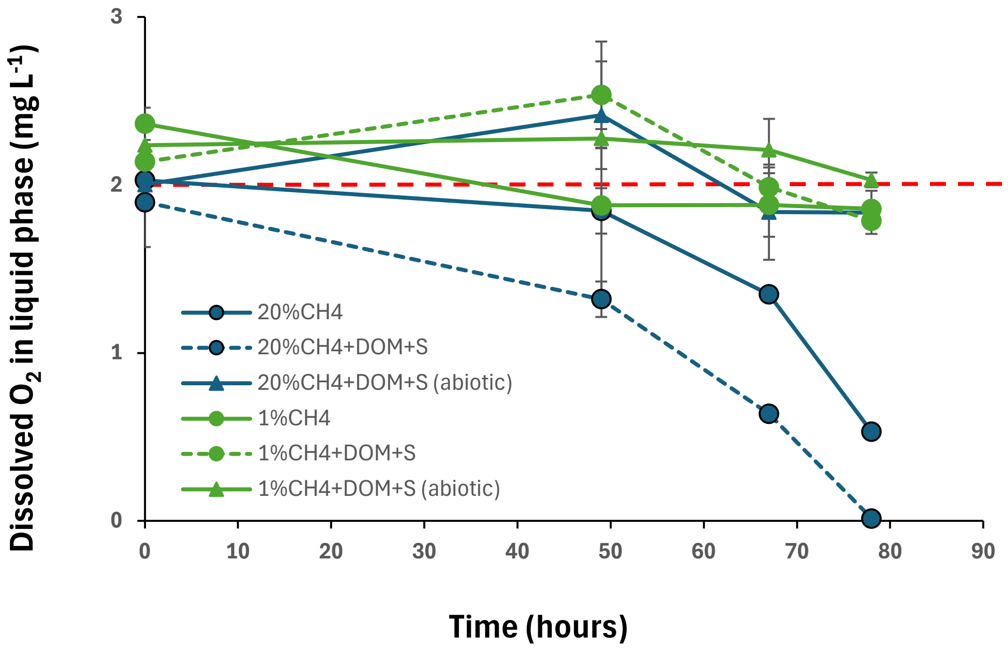

Figure S4. Dissolved O_2 concentration in the liquid phase during the experiment testing the effect of dissolved organic matter (DOM) and sulfide (S) on CH_4 metabolism of *Methylobacter* sp. S3L5C at two different CH_4 levels (1% and 20% in headspace). There was only one bottle measured per each treatment. The averages and deviations represent those of technical replicates. The vertical dashed red line represents upper measurement limit of the O_2 sensor (measurement range 0 – 2 mg L^{-1}). O_2 was not measured in treatments with CH_4 and DOM without sulfide. However, based on the existing data we expect that also in that treatment O_2 was consumed more rapidly at 20% CH_4 than at 1% CH_4.
